# Turning over New Leaves

**Published:** 1889-07-20

**Authors:** 


### Turning Over New Leaves.

(Books for Review should be sent to The Editor, The Lodge, Porchester Square, W.)

### MR. FROUDE AS A NOVELIST.*

The advice given us in our childhood to do one thing at a time, and that thing well, seems in many minds to develop with increasing years into a conviction that no one can do more than one thing passably. Mr. Froude is a historian, a sucessful historian, as even his enemies must admit, though some incline to the notion that the pen which makes the life of the past stand clear and vivid before us must, because it does so, be of necessity destitute of the critical acumen that can give a cool and balanced judgment on bygone actions and policies. They complained that the author of "The English in Ireland in the Eighteenth Century" was a romancer turned historian. They say now that the author of "The Two Chiefs of Dunboy" is an historian turned romancer, and failing in his self-appointed task. There's no pleasing some people! But if to lavish picturesque writing on a history be a crime in the eyes of the dry-as-dusts, surely, to put a flavour of history into an historical romance is at worst a misfortune. How was it to be avoided! Our æsthetic souls may be ready to gratify Mr. Oscar Wilde's taste for anachronisms; but even he would not recommend a novel that contained nothing but anachronisms. We must have a little leaven of truth, if only to prove the superiority of fiction.

Having read several reviews of "The Two Chiefs of Dunboy" before we took up the work itself, we expected to find it something like many historical novels we wot of—a stalking-horse for one man's opinions, a stage on which modern men and women masquerade in old-fashioned garments, unable, in spite of their "i' faiths," "gramercys," and "by'r ladys," to convince anyone that they are even the ghosts of the mediævals they personate. On the contrary, we found a book which, we venture to say, would have won almost unmixed praise from critics if it had been given to the world as a newly-discovered work of Sir Walter Scott. A book, the vigour and brilliancy of which would have caused almost hysterical rejoicing if it had come from the pen of Mr. Louis Stevenson. But coming from Mr. Froude—Froude the historian, the traveller, the biographer—the historical element in it has been used to imply that as a romance it is a failure.

Here and there, it is true, Mr. Froude does betray the fact that he is not a professional novelist—a manufacturer of three volumes per annum. Occasionally, he puts into the mouth of one of his characters a historical retrospect which might more conveniently be told by the author *in propria persona*. Perhaps he knew that the average reader skips description, but we do not skip Mr. Froude. Such vivid word-painting as his, such beauty of style, are things for which we can only be grateful. And it can hardly be denied that in the two chiefs—Morty Sullivan, the proscribed Irishman, and Colonel Goring, the English landowner—Mr. Froude has drawn two noble and heroic characters. Neither is perfect; race prejudices and the very desire each feels in different ways to do the best he can for that corner of Ireland to which both lay claim, prevent their appreciating each other's worth; but the reader, standing apart, can admire and sympathise with both, and he must feel that it is the true instinct of tragedy that sets two such men at lifelong feud with each other.

"The Two Chiefs of Dunboy" is in no sense a partisan book. It exposes equally the alternate weakness and cruelty of the English Government and the restless instability that mars so much that is good in the Irish character. He condemns unsparingly the use of agrarian outrage as a form of warfare; but he condemns also the narrow-minded selfishness that ruined Irish industries to catch English votes. But, just because it is so mercilessly just to both parties, it will give whoever reads it a clearer idea of the fundamental quarrel between England and Ireland than would listening to all the speeches of all the politicians. And we think also that it will convince the reader that the salvation of Ireland depends less on the acts of any Parliament, either in London or Dublin, than on the efforts made by such men as Goring in Mr. Froude's novel, and renewed by one or two men and women of our day, to develop Irish commerce and industry. There is no more keen-witted nation in the world than the Irish. They are naturally intelligent and naturally industrious, but a combined feeling of obstinacy and sentiment makes them often prefer old ways to new. If their industry and intelligence could be turned from the thankless task of tilling barren land, and the still more thankless work of political agitation, and directed into new and more hopeful channels, it is more than probable that the Irish question would settle itself.

* "The Two Chiefs of Dunboy: An Irish Romance of the Last Century." By J. A. Froude. London. Longmans, Green, and Co. Price 6s.

